# Significance as a Prognostic Factor of Eosinophil Count in Nasal Polyp Tissue in Patients with Chronic Rhinosinusitis Accompanied by Asthma

**DOI:** 10.3390/jcm13195849

**Published:** 2024-09-30

**Authors:** Moo Keon Kim, Seok Hyun Cho, Ha Na Lee, Seon Min Jung, Jin Hyeok Jeong

**Affiliations:** Department of Otolaryngology-Head & Neck Surgery, Hanyang University College of Medicine, Seoul 11923, Republic of Korea

**Keywords:** rhinosinusitis, eosinophil, asthma

## Abstract

**Background/Objectives:** Patients with chronic rhinosinusitis (CRS) accompanied by asthma often show poor prognoses and require continuous management. This study aimed to assess the prognostic value of eosinophil counts in nasal polyp tissue for selecting individuals who would benefit from ongoing management in CRS patients with asthma. **Methods:** Patients with asthma who underwent endoscopic sinus surgery for CRS with nasal polyps were included in the study. Eosinophil counts in nasal polyp tissues were quantified, and retrospective data were collected from laboratory and clinical findings, including endoscopic examinations, CT scans, and Japan Endoscopic Sinus Surgery Rating and Evaluation Committee (JESREC) scores. Disease control status was evaluated through endoscopic examination 6 months post-surgery. **Results:** A total of 42 patients were divided into two groups based on their disease management status 6 months post-operation: the well-control group (24 patients, 57.14%) and the poor-control group (18 patients, 42.86%). Demographics and laboratory findings did not show significant differences between the groups. However, the JESREC score (*p* = 0.04) and tissue eosinophil count (*p* = 0.02) were significantly different. Multivariate analysis identified tissue eosinophil count as the only risk factor associated with prognosis, with a cut-off value of 90/HPF. **Conclusions:** In CRS patients with asthma, high tissue eosinophil counts in nasal polyps were associated with poor disease control, which is the most potent predictor of prognosis. The assessment of eosinophil counts in nasal polyp tissue could aid in identifying patients who would benefit from continuous management and tailored interventions for improved outcomes.

## 1. Introduction

Chronic rhinosinusitis (CRS) is a persistent inflammatory disorder affecting the nasal mucosa and shows various manifestations. Diverse immune cells and their associated inflammatory mediators coordinate the wide-ranging spectrum of CRS [1]. Chronic rhinosinusitis without nasal polyps (CRSsNP) is generally characterized by predominantly neutrophilic inflammation, accompanied by increased levels of T helper 1 (Th1) cytokines. In contrast, chronic rhinosinusitis with nasal polyps (CRSwNP) is often associated with eosinophilic inflammation and elevated levels of Th2 cytokines [2,3]. Identifying the inflammatory patterns in CRS not only enhances our understanding of pathophysiology but also helps in choosing appropriate treatment approaches [4,5]. This is why there has been a shift towards classifying CRS based on endotypes rather than phenotypes.

One of the characteristic features of Th2 inflammation is blood and tissue eosinophilia. Tissue eosinophilia in CRS is generally linked to extensive sinus disease, worse post-operative outcomes, less improvement in disease-related quality of life, and higher recurrence rate [5,6,7]. Owing to the significance of tissue eosinophilia in CRS prognosis, CRS was recently classified based on tissue eosinophilia as eosinophilic CRS (ECRS) and non-ECRS [6,8]. Tokunage T et al., suggested diagnostic criteria of ECRS using preoperative findings [9]. The variables of the diagnostic criteria were bilateral CRS, nasal polyps, ethmoidal predominance on paranasal CT, and elevated level of eosinophils in peripheral blood. They assigned scores to each variable and proposed that if the total score, which was nominated as the “JESREC score”, exceeded 11 points, CRS could be diagnosed as ECRS. Although JESREC score has widely been accepted, the gold standard of diagnosing ECRS is the histopathological examination of nasal tissue [10,11]. Nevertheless, consistent diagnostic criteria of tissue eosinophil count for ECRS have not been firmly established. Furthermore, there have been few studies that have provided clear threshold values of eosinophil count associated with overall prognosis.

Among various comorbidities that have a negative impact on the prognosis of CRS, asthma is the most prominently mentioned comorbidity. Both asthma and CRSwNP display similar physio-pathological features of inflammation, such as airway remodeling and increased levels of type2 inflammatory cytokines (IL-4, IL-5, and IL-13) [12,13]. Therefore, patients with both CRSwNP and asthma often show more severe disease conditions and higher recurrence rates [13,14]. In many studies analyzing the risk factors related to refractory rhinosinusitis, asthma has been mentioned as a representative risk factor [15,16]. Tokunaga T et al. revealed that CRS patients with asthma showed clinical courses like moderate to severe CRS [9]. They analyzed that the recurrence rate was from 31% to 52% in asthmatic CRS patients. Gill et al. reported that risk of revision ESS was twice as high in patients with asthma, and especially in CRSwNP, the frequency was six times higher [17]. Zhang et al. reported that even after extensive ESS up to the Draf III method, the 5-year recurrence rate was high as 95.6–96.1% in asthmatic patients [15]. Therefore, the prognostic factor in asthma patients might be different and needs to be analyzed separately from other general CRS cases. While there are numerous studies on the prognostic factors in general CRS, there is a lack of research on the prognostic factors in CRS coexisting with asthma. In general CRS, preoperative CT scan and endoscopic findings are known to be related to prognosis [9]. However, in asthma patients, the preoperative condition is mostly severe, which limits preoperative findings’ use as prognostic factors.

In this study, we aimed to investigate the clinical impact of eosinophil count in nasal polyp tissue in CRS patients accompanied by asthma. We analyzed which clinical factors were associated with prognosis and showed the significance of eosinophil count in nasal polyp tissue as the prognosis factor. Moreover, we determined the clear cut-off value of eosinophil count that shows a difference in the post-operative prognosis.

## 2. Materials and Methods

### 2.1. Subjects

This study was conducted on CRSwNP patients who had coexisting asthma and underwent endoscopic sinus surgery (ESS). We retrospectively analyzed charts from 2010 to 2022 and selected cases that met all of the following inclusion criteria: (1) all preoperative records, including blood tests, paranasal CT scans, symptom scores, and endoscopic nasal findings were available; (2) histopathological examination were performed on nasal polyp tissue during surgery; (3) attended outpatient visits for more than six months after ESS. Patients with insufficient records or unclear evidence of coexisting asthma were excluded. This study was approved by the Institutional Review Board (IRB) of Hanyang University Guri Hospital (Approval No. 2023-08-018, approval date 18 August 2023).

### 2.2. Preoperative Evaluation

We collected demographic characteristics, including gender, age, height, weight, smoking and alcohol consumption status, underlying diseases, and the surgical history. For evaluating preoperative status, we collected endoscopic findings, paranasal CT findings, and blood test results. To evaluate preoperative disease status, endoscopic findings were recorded using the modified Lund–Kennedy (LK) endoscopic score ([18], Table A1), and CT findings were scored using the Lund–Mackay (LM) staging system ([19], Table A2). They were all evaluated and scored by one otolaryngologist.

Blood test results included peripheral blood white cell counts, eosinophil absolute counts and ratios, and total immunoglobulin (Ig) E levels. We assessed patients’ symptoms and disease-related quality of life using the Sino-Nasal Outcome Test (SNOT-22) questionnaire [20]. A high SNOT-22 score indicates more severe symptoms due to the disease. Additionally, we evaluated the preoperative olfactory status of patients using the Korean Version of Sniffin Sticks (KVSS) II test [21]. A low KVSS II test score means that the patient’s sense of smell is poor. Finally, we comprehensively assessed the patients’ condition and scored their preoperative status according to the Japan Epidemiological Survey of Refractory Eosinophilic Chronic Rhinosinusitis (JESREC) scoring system ([9], Table A3).

### 2.3. Evaluation the Eosinophil Count of Nasal Polyp Tissue

All patients underwent ESS under general anesthesia by one otolaryngologist. The polyp tissue samples were fixed in 4% paraformaldehyde at 4 °C for three days, dehydrated through alcohol, and then processed into paraffin wax blocks. The paraffin blocks were cut into 4 μm thickness and underwent staining with hematoxylin and eosin (H&E). The eosinophil count was evaluated in the lamina propria at 400× magnification under an optical microscope. Eosinophil counts per high power field (HPF) were calculated for four random fields in each section, and the average value was defined as the final tissue eosinophil count (TEC, eosinophils/HPF).

### 2.4. Postoperative Care and Evaluation

Patients were regularly followed up through outpatient visits after surgery. During the first month, they visited the outpatient department weekly for nasal dressings, and thereafter, they visited the outpatient department at one-month intervals to assess the nasal condition and perform nasal dressings if needed. For the first three weeks after surgery, patients took oral antibiotics, followed by one week of oral steroids (methylprednisolone). Subsequently, patients maintained nasal saline irrigation without regular medication and continued with periodic follow-up. If the disease was not well controlled, the frequency of outpatient visits was increased, and antibiotics and steroids were prescribed as needed. At the six-month follow-up outpatient visit after surgery, the degree of disease control was recorded. Nasal endoscopic findings were documented using the modified LK endoscopic score, same as the preoperative evaluation. Furthermore, we assessed the disease control status of CRS into controlled, partially controlled, and uncontrolled groups according to the European Position Paper on Rhinosinusitis (EPOS) 2020 guideline ([22], Table A4). Based on this classification, we defined the controlled group as the “well-control group”, while we defined partially controlled and uncontrolled group as the “poor-control group”.

### 2.5. Statistical Analysis

All continuous variables were expressed as mean ± standard deviation values. We compared each variable between well-control group and poor-control group and analyzed which variable was related to CRS prognosis. The independent *t*-test and χ^2^ test were employed for analysis. Multivariate analysis was performed using logistic regression for finding variables associated with prognosis. Furthermore, we plotted ROC curves for significant factors, and the values of area-under-curve (AUC) were calculated to evaluate their diagnostic value. To determine the accurate cut-off values for each factor, we used the Youden index, setting it to the value at which (sensitivity + specificity-1) was maximized.

A *p*-value of less than 0.05 was defined as statistically significant, using IBM SPSS statistics version 28.0 software (IBM, SPSS Inc., Somers, NY, USA).

The group definition in our study according to EPOS 2020 is as follows.


**Group**

**Disease Status 6 Months after Surgery According to EPOS 2020**
Well-controlControlledPoor-controlPartly controlled or Uncontrolled

## 3. Results

A total of 42 patients were enrolled in the study. We divided patients into two groups based on their disease management status at 6 months after ESS as described in the methods section: the well-control group and poor-control group (Table A4). The well-control group consisted of 24 individuals (57.14%), while the poor-control group had 18 individuals (42.86%). When evaluated according to the EPOS criteria, 16 out of 18 patients (88.9%) in the poor control group reported nasal blockage, 13 patients (72.2%) reported rhinorrhea, and 3 patients (16.7%) complained of facial pain. A total of 11 patients (61.1%) reported hyposmia, while 6 patients (33.3%) complained of fatigue. Diseased mucosa was observed in 17 patients (94.4%) on nasal endoscopy, and rescue treatment was used in 16 patients (88.9%). There was no significant difference in the demographics between the two groups (Table 1). We compared whether there were differences in the preoperative findings between two groups (Table 2). The laboratory findings, including WBC and eosinophil count in peripheral blood and IgE value, did not show significant differences. Furthermore, there were no differences in preoperative endoscopic findings, CT findings, and symptom scores. On the other hand, there were significant differences in JESREC scores and TEC. The JESREC score in the poor-control group was 14.94 points, which was significantly higher than well-control group (12.83 points) (*p* = 0.04). TEC was also significantly higher in poor-control group (124.22/HPF) than in the well-control group (61.88/HPF) (*p* = 0.02).

We further underwent multivariate analysis to find the risk factors related to prognosis (Table 3). Among many variables, TEC was the only significantly associated factor (*p* = 0.03). Other factors, including the JESREC, score did not show statistical significance.

### TEC as a Prognostic Factor

**Figure 1:**
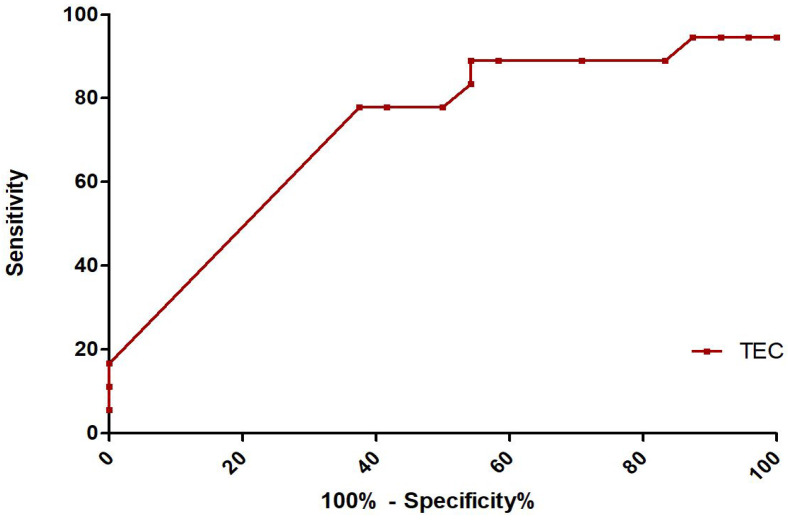
ROC (receiver operating characteristic) curve for prognosis of tissue eosinophil count (TEC) The value of area under curve (AUC) in TEC was 0.723. Using Youden’s index, the cut-off value of TEC that determined prognosis was 90/HPF.

We further analyzed the significance of TEC as a prognosis factor. An ROC curve was plotted for TEC, for which the AUC was 0.723 (Figure 1). We determined the cut-off value of TEC for prognosis using the Youden index, which was 90/HPF (sensitivity 77.78% and specificity 62.50%).

We further divided the patients into two groups based on a TEC value of 90/HPF, defining those with TEC equal to or above 90 as the ‘high-TEC’ group and those with values below 90 as the ‘low-TEC’ group. The high-TEC group had 23 individuals (54.76%), and the low-TEC group had 19 individuals (45.24%). The demographics and preoperative findings were analyzed between the two groups (Table 4). Only gender distribution showed a significant difference, in which the high-TEC group had a higher proportion of females (*p* = 0.04). Other variables, including age, BMI, and preoperative findings, did not show significant differences.

We further compared the post-operative status six months after ESS to analyze prognoses between high- and low-TEC groups (Table 5). In the high-TEC group, the LK endoscopy score six months after surgery was significantly higher at 1.87 compared to 0.47 in the low-TEC group (*p* = 0.002). Additionally, in the high-TEC group, the number of patients whose disease was poorly controlled was significantly higher at 14 (60.9%) (*p* = 0.01). The calculated odds ratio was 5.83 (95% CI 1.46–23.30). On the other hand, we found that there was no significant difference in the amounts of steroids or antibiotics used during the six months after surgery between the two groups.

## 4. Discussion

The prognosis of CRS has been studied for a long period through numerous studies. A challenge for predicting CRS prognosis has been that because no singular significant factor has been defined. Radiographic severity; endoscopic severity; preoperative biomarkers such as IL-4, IL-5, IL13, and IgE; or subjective symptoms have all been suggested as the prognostic factors of CRS. Bai J et al. evaluated the prognostic association of various clinical variables in 94 CRSwNP patients after sinus surgery [23]. They found that polyp recurrence had strong relationships with radiologic and endoscopic severity, the comorbidity of asthma, and patients’ symptom scores. Not only this study but also numerous other studies have suggested similar prognostic factors [22,24]. Therefore, it has been generally accepted that the various factors significantly influence the clinical courses and prognosis of CRS, sparking interest in the endotype of CRS. Lou et al. focused on the disease recurrence in each cellular phenotype of NP [25]. They revealed that plasma cells or lymphocytes in dominant-type CRSwNP showed less than 7% of recurrence rates, and neutrophilic CRSwNP had recurrence rates of 46.4%. Through various other studies, type 2 inflammation with the infiltration of eosinophils in nasal mucosal tissues has been highlighted as an important point, leading to various studies in ECRS.

The gold standard of defining ECRS is measuring the eosinophil density in sinus mucosa on H&E stain. However, there is no universally accepted diagnostic value for distinguishing between ECRS and non-ECRS. TEC 70/HPF has widely been used as a criterion of ECRS in many studies [26], but some studies have presented different diagnostic criteria of TEC. Kim et al. conducted a meta-analysis of ECRS papers, demonstrating that a wide range of TEC values were used as criteria for ECRS, ranging from 5 to 120 eosinophils/HPF [27]. However, some values were only used in one or two papers, and some researchers set the cut-off values up based on their own criteria. Furthermore, some research analyzed only CRSwNP, while others encompassed both CRSsNP and CRSwNP, leading to heterogeneity of study. Likewise, there are no clear histological criteria that definitively define ECRS so far, and the diagnosis of ECRS is based on a comprehensive consideration of blood tests, imaging studies, phenotype, and comorbidities.

Some studies have revealed the relationship between the recurrence of NP and tissue eosinophilia. Lou et al. observed distinct features in CRSwNP patients, focusing on the disease recurrence in each cellular phenotype of NP [25]. Patients with plasma cells or lymphocytes dominant type showed less than 7% of polyp recurrence. Patients with mixed inflammation or neutrophil infiltration had recurrence rates of 75% and 46.4%, respectively. CRSwNP dominated by eosinophils showed the highest polyp recurrence rate at 98.5%. Lou et al. also identified various factors that affect disease recurrence after ESS through further study [28]. The factors that predicted higher recurrence rates were comorbid asthma, symptom score, LM CT score, fractional exhaled nitric oxide, and mucosal and blood inflammatory cells. Multivariate analysis revealed that a tissue eosinophil proportion exceeding 27% and a tissue eosinophil absolute count exceeding 55/HPF were the most powerful predictors of NP recurrence after surgery. However, defining the exact eosinophil count in relation to prognosis could not be clearly defined.

Although the diagnostic value of ECRS and the significance of tissue eosinophilia as a prognostic factor in CRS has been widely explored, all of these studies have targeted general CRS patients. CRS patients with asthma have distinct characteristics compared to typical CRS, and their clinical outcomes are generally poor. CRS with asthma patients showed more severe symptoms and recalcitrant clinical courses, such as a low response to medical treatment and high recurrence rate after surgery [29,30]. In many studies analyzing the risk factors of refractory rhinosinusitis, asthma has been mentioned as a representative risk factor [15]. Our study has significant value in that we targeted CRSwNP patients accompanied by asthma after surgery. We aimed to analyze the factors that influence clinical outcomes in asthmatic CRS, show the significance as the prognostic factor of TEC, and tried to find precise cut-off value. A TEC of 90/HPF was confirmed to predict the control of disease with a sensitivity of 77.78% and a specificity of 62.50%. Because we selected the asthma patients, the cut-off value of TEC in our study was higher than other studies [27,28].

Our study also calculated and analyzed the JESREC score in each patient. The JESREC score is a scoring system based on the risk of disease recurrence after ESS suggested by Tokunaga T et al. in 2015 [9]. They defined that cut-off value for ECRS as 11 points, and the maximum score is 17 points. They also pointed out that beyond the JESREC score, the heterogeneity of CRS makes it difficult to diagnose refractory CRS. They analyzed that even in the high-JESREC-score group, the clinical course was more severe and refractory in patients with asthma, aspirin intolerance, and NSAID intolerance. Our study targeted the asthma patients that generally showed high JESREC scores and refractory clinical courses. In our study, the average JESREC score was 13.74 ± 3.37 points, and 36 patients (85.71%) scored 11 or higher. Moreover, 15 patients (35.7%) scored 15 points, and 12 patients (28.6%) scored 17 points, which is the maximum score. We also tried to define the cut-off value of JESREC score and determined the value as 16 points, which is a near-perfect value. These results showed that the JESREC score has limitations as a prognostic factor in asthma patients. Furthermore, we revealed that only TEC is a significant prognostic factor through the multivariate analysis.

Although our study showed a significant prognostic value of TEC, there were several limitations. First of all, the number of patients was relatively small, with a total of 42 individuals, due to the precise selection of CRS patients coexisting with asthma. The limited size of the population studies may preclude the use of only the TEC as the cut-point for deciding on more aggressive post-op care, including biologics. A large study population should be considered. Secondly, we assessed the disease control based on 6-month status after surgery, which is insufficient to assess long-term prognosis. It would be ideal to perform follow-up observations for a longer period in order to analyze the long-term prognosis of TEC. The problem was that most patients tended to have good compliance with outpatient visits up to 6 months after surgery, regardless of the disease control level, but as time progressed, there was a decrease in outpatient visits, especially among well-controlled patients. This can be an important bias of the study. In our study, only two patients (4.55%) did not make outpatient visits at the 6-month follow-up, who were excluded from the subjects. However, at the one-year follow-up, eight patients (19.05%) did not attend outpatient visits, and among them, seven were those whose disease was well controlled at the 6-month follow-up. If the total number of patients increases in the future, we believe that a long-term analysis of the study can be conducted without bias.

We demonstrated that the TEC value itself could be used as an indicator of prognosis, especially in patients strongly suspected of having type 2 inflammation and refractory CRS. Additionally, it was revealed that the high-TEC group exhibited poorer disease control and endoscopic findings after surgery, even though their clinical characteristics did not significantly differ from those in the low-TEC group. Patients with high TEC should be observed more thoroughly and required more aggressive treatment. Recently, biologics have been considered as a new treatment option for refractory CRS. However, due to their high cost, there is much debate about the appropriate patient selection. The marked difference in low TEC and high TEC post-op at 6-month follow-up strongly suggests, although limited by the number of subjects studied, that biologics should be considered in the high-TEC group if further reports duplicate our findings. Furthermore, it is believed that various therapeutic approaches can be considered for high-TEC patients.

## Figures and Tables

**Table 1:** Demographics.

	Well-Control (*n* = 24)	Poor-Control (*n* = 18)	Total (*n* = 42)	*p*
Gender (M:F)	10:14	9:9	19:23	0.41
Age (years)	51.54 ± 13.38	49.39 ± 12.35	50.62 ± 12.84	0.59
BMI, kg/m^2^	24.36 ± 3.11	24.80 ± 3.30	24.55 ± 3.16	0.66
Alcohol (*n*, %)	6 (25.0%)	3 (16.7%)	9 (21.4%)	0.40
Smoking (*n*, %)	6 (25.0%)	7 (38.9%)	13 (31.0%)	0.27
Hypertension (*n*, %)	8 (33.3%)	4 (22.2%)	12 (28.6%)	0.43
DM (*n*, %)	5 (20.8%)	1 (5.6%)	6 (14.3%)	0.17
History of sinus surgery (*n*, %)	3 (12.5%)	3 (16.7%)	6 (14.3%)	0.52

**Table 2:** Preoperative findings in the well-control and poor-control groups.

	Well-Control	Poor-Control	*p*
WBC count (/mm^3^)	6870.83 ± 1692.31	7205.56 ± 1886.63	0.55
Eosinophil percentage (%)	7.05 ± 3.96	9.11 ± 6.11	0.19
Eosinophil count (/mm^3^)	476.20 ± 290.28	621.23 ± 380.62	0.16
Total IgE (IU/mL)	328.32 ± 302.71	376.50 ± 341.34	0.63
LK endoscopic score	6.42 ± 2.50	5.11 ± 2.22	0.09
LM CT score	16.38 ± 5.41	17.28 ± 4.60	0.57
SNOT-22 score	37.67 ± 22.22	41.06 ± 27.20	0.66
KVSS II score	17.29 ± 10.61	13.39 ± 8.21	0.20
JESREC score	12.83 ± 3.63	14.94 ± 2.62	0.04 *
Tissue eosinophil count (/HPF)	61.88 ± 35.11	124.22 ± 113.49	0.02 *

* Statistically significant difference. Abbreviation: BMI, body mass index. DM, diabetes mellitus. LK, Lund–Kennedy. LM, Lund–Mackay. SNOT, Sinonasal Outcomes Test. KVSS, Korean Version of Sniffin Sticks. JESREC, Japanese Epidemiological Survey of Refractory Eosinophilic Chronic Rhinosinusitis.

**Table 3:** Risk factors associated with prognosis.

	OR (95% CI)	*p*
Gender	0.70 (0.20–2.44)	0.57
Age	0.98 (0.93–1.03)	0.46
BMI	1.07 (0.86–1.32)	0.55
Smoking	0.27 (0.03–2.15)	0.21
Alcohol	3.59 (0.59–21.84)	0.17
DM	0.09 (0.01–1.41)	0.09
History of sinus surgery	3.89 (0.32–47.00)	0.29
WBC count	1.00 (1.00–1.001)	0.37
Eosinophil ratio	1.23 (0.76–1.99)	0.39
Eosinophil count	0.99 (0.99–1.01)	0.60
Total IgE	1.003 (0.99–1.01)	0.11
LK endoscopic score	0.68 (0.99–1.001)	0.15
LM CT score	1.11 (0.87–1.42)	0.40
SNOT-22 score	0.98 (0.93–1.04)	0.55
JESREC score	1.23 (0.91–1.64)	0.09
Tissue eosinophil count (/HPF)	1.02 (1.001–1.04)	0.03 *

* Statistically significant difference. Abbreviation: LK, Lund–Kennedy. LM, Lund–Mackay. SNOT, Sinonasal Outcomes Test. JESREC, Japanese Epidemiological Survey of Refractory Eosinophilic Chronic Rhinosinusitis.

**Table 4:** Demographic characteristics and preoperative findings between the high-tissue-eosinophil-count (TEC) and low-TEC group.

	High TEC (*n* = 23)	Low TEC (*n* = 19)	*p*
Gender (M:F)	7:16	12:7	0.04 *
Age (years)	48.61 ± 12.74	53.05 ± 12.88	0.27
BMI (kg/m^2^)	24.72 ± 3.17	24.35 ± 3.21	0.71
Smoking (*n*, %)	3 (13.0%)	6 (31.6%)	0.26
Alcohol (*n*, %)	6 (26.1%)	7 (36.8%)	0.52
DM (*n*, %)	3 (13.0%)	3 (15.8%)	1.00
History of sinus surgery (*n*, %)	3 (13.0%)	3 (15.8%)	1.00
WBC count (/mm^3^)	6991.30 ± 1862.53	7042.11 ± 1686.32	0.93
Eosinophil percentage (%)	9.19 ± 5.55	6.42 ± 3.95	0.08
Eosinophil count (/mm^3^)	608.69 ± 341.85	453.21 ± 348.39	0.14
Total IgE (IU/mL)	386.44 ± 312.02	303.62 ± 324.81	0.41
LK endoscopic score	5.74 ± 2.14	6.00 ± 2.83	0.74
LM CT score	17.83 ± 4.16	15.47 ± 5.79	0.13
SNOT-22 score	42.91 ± 26.50	34.53 ± 20.92	0.27
TDI (KVSS II) score	13.96 ± 8.35	17.69 ± 11.10	0.23
JESREC score	14.57 ± 2.74	12.74 ± 3.84	0.08

* Statistically significant difference. Abbreviation: LK, Lund–Kennedy. LM, Lund–Mackay. SNOT, Sinonasal Outcomes Test. TDI, Threshold–Discrimination–Identification. KVSS, Korean Version of Sniffin Sticks. JESREC, Japanese Epidemiological Survey of Refractory Eosinophilic Chronic Rhinosinusitis.

**Table 5:** Differences in prognosis (6 months after surgery) between the high-tissue-eosinophil-count (TEC) and low-TEC group.

	High TEC	Low TEC	*p*
LK endoscopy score (6 months after surgery)	1.87 ± 1.84	0.47 ± 0.77	0.002 *
Poor control (*n*, %)	14 (60.9%)	4 (21.1%)	0.01 *
Usage of steroid (*n*, %)	7 (30.4%)	4 (21.1%)	0.73
Usage of antibiotics (*n*, %)	6 (26.1%)	2 (10.5%)	0.26

* Statistically significant difference. Abbreviation: LK, Lund–Kennedy.

## Data Availability

Data are contained within the article.

## Appendix A

**Table A1:** Modified Lund–Kennedy (LK) endoscopic score.

Endoscopic Findings	Nasal Cavity	
	Right	Left
Polyp (0, 1, 2)		
Edema (0, 1, 2)		
Secretion (0, 1, 2)		
Total		

Note: Polyp: 0—absent; 1—limited to the middle meatus; 2—extending to the nasal cavity. Mucosa edema: 0—absent; 1—mild/moderate edema; 2—polypoid degeneration. Secretion: 0—absent; 1—hyaline; 2—thick and/or mucopurulent.

**Table A2:** Lund–Mackay score of CT scan.

Paranasal Sinus CT	Right	Left
Maxillary (0, 1, 2)		
Anterior Ethmoid (0, 1, 2)		
Posterior Ethmoid (0, 1, 2)		
Sphenoid (0, 1, 2)		
Frontal (0, 1, 2)		
Ostiomeatal Complex (0, 2) *		
Total		

Note: 0—without abnormalities; 1—partial opacification; 2—total opacity. * 0—no obstruction; 2—obstructed.

**Table A3:** JESREC (Japanese Epidemiological Survey of Refractory Eosinophilic Chronic Rhinosinusitis) scoring system: Determining eosinophilic chronic rhinosinusitis (ECRS) and non-ECRS.

Factor	Score
Disease side: both	3
Nasal polyp	2
CT shadow: ethmoid greater than or equal to maxillary	2
Eosinophils in peripheral blood	
>2% but ≤5%	4
>5% but ≤10%	8
>10%	10
**Diagnosis**	**Total Score (JESREC Score)**
ECRS	≥11
Non-ECRS	≤10

**Table A4:** EPOS (European Position Paper on Rhinosinusitis and Nasal Polyps) 2020 guideline: Assessment of current clinical control of chronic rhinosinusitis.

	Controlled (All of the Following)	Partly Controlled (at Least 1 Present)	Uncontrolled (3 or More Present)
Nasal blockage	Not present or not bothersome	Present on most days of the week	Present on most days of the week
Rhinorrhea/Postnasal drip	Little and mucous	Mucopurulent on most days of the week	Mucopurulent on most days of the week
Facial pain/Pressure	Not presentor not bothersome	Present on most days of the week	Present on most days of the week
Smell	Normal or only slightly impaired	Impaired	Impaired
Sleep disturbance or fatigue	Not present	Present	Present
Nasal endoscopy	Healthy or almost healthy mucosa	Diseased mucosa	Diseased mucosa
Rescue treatment(in last 6 months)	Not needed	Need of 1 course of rescue treatment	Symptoms persist despite rescue treatment(s)
